# Median nerve electrical stimulation promotes myelin regeneration through regulating miR-1-3p/BDNF/TrkB-MAPK pathway in a transient cerebral ischemia rat model

**DOI:** 10.3389/fnbeh.2026.1820913

**Published:** 2026-09-11

**Authors:** Rui Li, Jingyi Lu, Chengcai Zhang, Xiaowei Zhang, Meiqi Wang, Ping Zhang, Kunli Yang, Hongmei Fang, Ying Gao, Kistina Mohamed, Che Ismail Che Noh, Yi Li, Kai Ling Chin

**Affiliations:** 1 Department of Rehabilitation Medicine, The Second People’s Hospital of Kunming, Rehabilitation Hospital Affiliated to Kunming University, Kunming, China; 2 Department of Biomedical Sciences, Faculty of Medicine and Health Sciences, Universiti Malaysia Sabah, Kota Kinabalu, Malaysia; 3 Yuxi Vocational and Technical College, Yuxi, China

**Keywords:** BDNF/TrkB-MAPK, ischemic stroke, median nerve electrical stimulation, miR-1-3p, myelin regeneration

## Abstract

**Introduction:**

Median nerve electrical stimulation (MNES) has emerged as a promising peripheral neuromodulatory strategy for improving motor recovery after ischemic stroke. Because the median nerve provides strong afferent input to central sensorimotor pathways, MNES may influence post-stroke neural repair beyond symptomatic motor modulation. However, the molecular mechanisms linking MNES to myelin repair remain unclear. Brain-derived neurotrophic factor (BDNF) is closely involved in neuronal survival, plasticity, and myelin remodeling, and bioinformatic analysis identified BDNF as a putative target of miR-1-3p. Therefore, this study investigated whether MNES-associated neurological recovery and myelin repair after ischemic stroke involve modulation of the miR-1-3p/BDNF/TrkB-MAPK pathway.

**Methods:**

Adult male Sprague-Dawley rats were subjected to middle cerebral artery occlusion/reperfusion (MCAO/R), followed by MNES every other day for seven sessions. Neurological function, infarct injury, neuronal survival, neuroinflammation, myelin integrity, and pathway-related molecular changes were evaluated using behavioral, histological, ultrastructural, RT-qPCR, and Western blot analyses.

**Results:**

MNES was associated with improved neurological performance, reduced infarct injury, better preservation of myelin structure, decreased miR-1-3p expression, and increased BDNF, TrkB, and MAPK expression. In miR-1-3p modulation experiments, miR-1-3p antagomir partially mimicked the neuroprotective and myelin-related effects observed after MNES, whereas miR-1-3p agomir attenuated several MNES-associated benefits and reduced BDNF/TrkB-MAPK pathway-related protein expression.

**Conclusion:**

These findings suggest that MNES may support early neurological recovery and myelin repair after ischemic stroke, at least in part through involvement of the miR-1-3p/BDNF/TrkB-MAPK pathway. Further studies are required to confirm direct molecular targeting, long-term efficacy, safety, and translational relevance.

## 1 Introduction

Upper limb dysfunction affects 70%–80% of stroke patients, and many do not achieve full recovery. Even 6 months post-stroke, 30%–50% of patients still suffer from severe upper limb impairment (Campbell and Khatri, 2020). Rehabilitation strategies aim to improve patient’s quality of life by facilitating motor recovery and promoting neural repair. Various therapies, such as robot-assisted therapy, constraint-induced movement therapy, and neuromuscular electrical stimulation, are used in clinical practice to restore upper limb function (Zhu et al., 2023). However, none of these therapies fully restore motor function in hemiplegic patients, highlighting the need for innovative treatment options (Chen et al., 2022). This dysfunction is primarily due to neuronal damage, which results in cell death, inflammation, and disrupted neural connections (Sekerdag et al., 2018). Despite the nervous system’s inherent self-repair mechanisms, the extent of natural recovery is limited by factors such as scar formation, inflammation, and ongoing neuronal death (Carmichael, 2006). Thus, there is a need for interventions that enhance and support neural repair to improve functional recovery, particularly in restoring motor functions and promoting neural plasticity.

Electrical stimulation has gained recognition as an early rehabilitation method for enhancing upper limb motor recovery by stimulating peripheral nerves and activating both motor and sensory fibers (Fernanda Silva et al., 2024). Among neuromodulatory approaches, median nerve electrical stimulation (MNES) has been shown to improve motor function by enhancing afferent input from the stimulated median nerve to central sensorimotor pathways, including the dorsal column nuclei, thalamus, and primary somatosensory and motor cortices, thereby promoting cortical reorganization and synaptic plasticity (Boakye et al., 2000; Klaiput and Kitisomprayoonkul, 2009; Quartarone et al., 2006). Consistent with this, functional near-infrared spectroscopy studies have demonstrated that MNES can induce sensorimotor activation and functional reorganization of distributed cortical networks following stroke (Huo et al., 2019). MNES is used clinically for patients in comas, stroke survivors, those with traumatic brain injuries, Parkinson’s disease, cognitive impairments, and movement disorders like Tourette syndrome and chronic tics (Iverson et al., 2023; Tsai et al., 2015; Wu et al., 2023; Zhou et al., 2024). Emerging clinical trials have demonstrated that MNES can enhance the impaired upper extremity movement function after stroke (Carrico et al., 2016; Shin et al., 2018) and improve wolf motor function test and action research arm test in patients with moderate-to-severe hemiparesis after stroke (Carrico et al., 2018; Shin et al., 2017). In a previous randomized clinical study, our group applied ultrasound-guided invasive MNES as a targeted neuromodulatory approach for stroke rehabilitation. This technique allows precise localization of the median nerve under real-time ultrasound guidance, thereby improving stimulation accuracy and reducing the risk of injury to surrounding tissues. Clinical findings from this study demonstrated significant improvement in upper limb motor function, particularly in the immediate period following the initial intervention (Li et al., 2022; Li R. et al., 2023). Despite the emerging clinical application of MNES in stroke rehabilitation, the underlying neuroprotective and neuroplastic mechanisms remain incompletely understood.

Myelin regeneration plays a critical role in restoring neurological function following ischemic stroke. Damage to oligodendrocytes, the primary cells responsible for myelin formation in the central nervous system, leads to myelin loss, axonal degeneration, and impaired neural signaling (Xie et al., 2021). Although spontaneous myelin regeneration can occur after stroke, it is often insufficient to achieve full functional recovery. Consequently, identifying strategies that promote oligodendrocyte proliferation, differentiation, and myelin repair remains a major focus and frontier in stroke research.

Brain-derived neurotrophic factor (BDNF) is a key neurotrophin involved in both neurodevelopment and neural repair following injury. BDNF is predominantly produced by excitatory neurons in the cortex and hippocampus but is also expressed by glial cells, including Schwann cells in peripheral nerves and oligodendrocyte lineage cells in the central nervous system, where it plays an important role in regulating myelination and axonal integrity. Following ischemic stroke, BDNF activates its high-affinity receptor, tropomyosin receptor kinase B (TrkB), thereby initiating signaling cascades that promote neuronal survival, synaptic plasticity, and neuronal growth (Alnoaman et al., 2025). Accumulating evidence has identified the BDNF/TrkB–mitogen-activated protein kinase (MAPK) signaling pathway as a critical regulator of oligodendrocyte differentiation and myelin formation, highlighting its potential as a therapeutic target for enhancing myelin repair (Fletcher et al., 2018).

Recent studies suggest that microRNAs (miRNAs) are important post-transcriptional regulators of neural injury and repair after ischemic stroke. By regulating target mRNAs, miRNAs can influence neuronal survival, neuroinflammation, glial responses, axonal remodeling, and myelin-related processes. Among these miRNAs, miR-1-3p has been widely studied in muscle regeneration (Schanda et al., 2022), cardiovascular diseases (Xu et al., 2022), cancer biology (Dai et al., 2023; Gao et al., 2018), and ischemic stroke (Benito et al., 2022). Its specific role in the brain after ischemic stroke remains insufficiently defined. Existing evidence suggests that miR-1-3p may participate in ischemic injury-related molecular responses, but the direction and functional significance of miR-1-3p changes may depend on the disease model, brain region, cell type, and post-injury stage. Therefore, miR-1-3p should not be regarded as a fully established regulator of ischemic stroke symptoms or recovery. Its potential contribution to post-stroke neural repair, especially myelin-related remodeling, requires further investigation.

In present study we predicted the potential target genes for miR-1-3p using the TargetScan database^1^ and identified BDNF mRNA as a putative target, suggesting a possible regulatory link between miR-1-3p and BDNF-mediated myelin repair after ischemic stroke. BDNF is a key neurotrophin involved in neuronal survival, synaptic plasticity, oligodendrocyte lineage regulation, and myelin remodeling. After binding to its high-affinity receptor TrkB, BDNF activates downstream signaling cascades, including MAPK-related pathways, which are closely associated with neural repair after ischemic injury. Previous studies have shown that peripheral nerve stimulation increases BDNF mRNA and protein levels in the stimulated nerves, spinal cord, and sensorimotor cortex, and that pharmacological blockade of BDNF/TrkB signaling attenuates these beneficial effects (Huie et al., 2012; Wenjin et al., 2011). Nevertheless, the underlying mechanism remains unclear. Thus, in this study, we verified the effects of MNES on myelin repair and neurological function recovery in rat models of ischemic stroke, and explored the undergoing mechanism. We found that MNES reduced the expression levels of miR-1-3p and enhanced the BDNF/TrkB–MAPK signaling pathway. Our findings indicate that MNES could activate the BDNF/TrkB-MAPK pathway through inhibiting the expression of miR-1-3p, enhancing myelin regeneration and improving the neuronal recovery post-stroke.

## 2 Materials and methods

### 2.1 Animals

Adult male Sprague-Dawley rats (*n* = 144, weighing 250 ± 20 g) were obtained from Hunan Sileke Laboratory Animal Co., Ltd. and housed under standard laboratory conditions with a 12-h light/dark cycle at the Animal Research Center of Yunnan University of Chinese Medicine. All experiments were conducted in compliance with the Guidelines for the Care and Use of Laboratory Animals (8th Edition) (National Research Council Committee for the Update of the Guide for the Care and Use of Laboratory Animals, 2011) and followed the ARRIVE reporting guidelines (Percie du Sert et al., 2020). The study protocol was approved by the Ethics Committee of Kunming Second People’s Hospital [Approval No. 2021 (06)] on 23 June 2021.

### 2.2 Experimental design and grouping

Rats were randomly assigned to eight experimental groups (*n* = 18 per group). A computer-generated random number list was used to generate the allocation sequence *a priori* (simple randomization; 1:1 allocation ratio across the eight groups). The group assignments were concealed in sequentially numbered, opaque, sealed envelopes, which were opened only after each animal had been enrolled. The Sham group served as the control and underwent identical surgical exposure without middle cerebral artery occlusion (MCAO). All other groups were subjected to transient left MCAO and reperfusion, including the MCAO group, MNES group, PBS (vehicle control) group, miR-1-3p antagonist (miR-1-3p-antago) group, miR-1-3p-antago + MNES group, miR-1-3p agonist (miR-1-3p-ago) group, and miR-1-3p-ago + MNES group. The miR-1-3p modulation groups were designed to evaluate the effects of miR-1-3p inhibition or upregulation on ischemic injury and to determine whether MNES exerts additive or counteracting effects under these conditions.

To accommodating multiple outcome assessments requiring incompatible tissue processing protocols, animals within each group were allocated to specific analyses. Six rats per group were used for infarct volume assessment using triphenyl tetrazolium chloride (TTC) staining; six rats were used for molecular and ultrastructural analyses, including Western blotting (WB), reverse transcription-quantitative polymerase chain reaction (RT-qPCR), and transmission electron microscopy (TEM); and six rats were used for histological analyses, including immunofluorescence (IF) staining, Luxol Fast Blue (LFB) staining, and Nissl staining. These analyses require distinct fixation, preservation, and sectioning procedures and therefore could not be performed on the same animals (Table 1).

**TABLE 1:** Experimental groups and sample allocation.

Group	Intracerebroventricular injection	MNES treatment	TTC staining (n)	WB/ RT-qPCR/TEM (*n*)	*IF/LFB/Nissl staining (*n*)	Total rats (*n*)
Sham	No	No	6	6	6	18
MCAO	No	No	6	6	6	18
MNES	No	Yes	6	6	6	18
PBS	PBS	No	6	6	6	18
miR-1-3p-antago	miR-1-3p antagonist	No	6	6	6	18
miR-1-3p-antago + MNES	miR-1-3p antagonist	Yes	6	6	6	18
miR-1-3p-ago	miR-1-3p agonist	No	6	6	6	18
miR-1-3p-ago + MNES	miR-1-3p agonist	Yes	6	6	6	18

*The same six rats allocated for histological analyses were used for myelin basic protein (MBP) immunofluorescence, Luxol fast blue (LFB), and Nissl staining. Three of these six rats were additionally selected for ionized calcium-binding adapter molecule 1 (Iba-1) and neuronal nuclei (NeuN) immunofluorescence analyses using adjacent brain sections.

**FIGURE 1:**
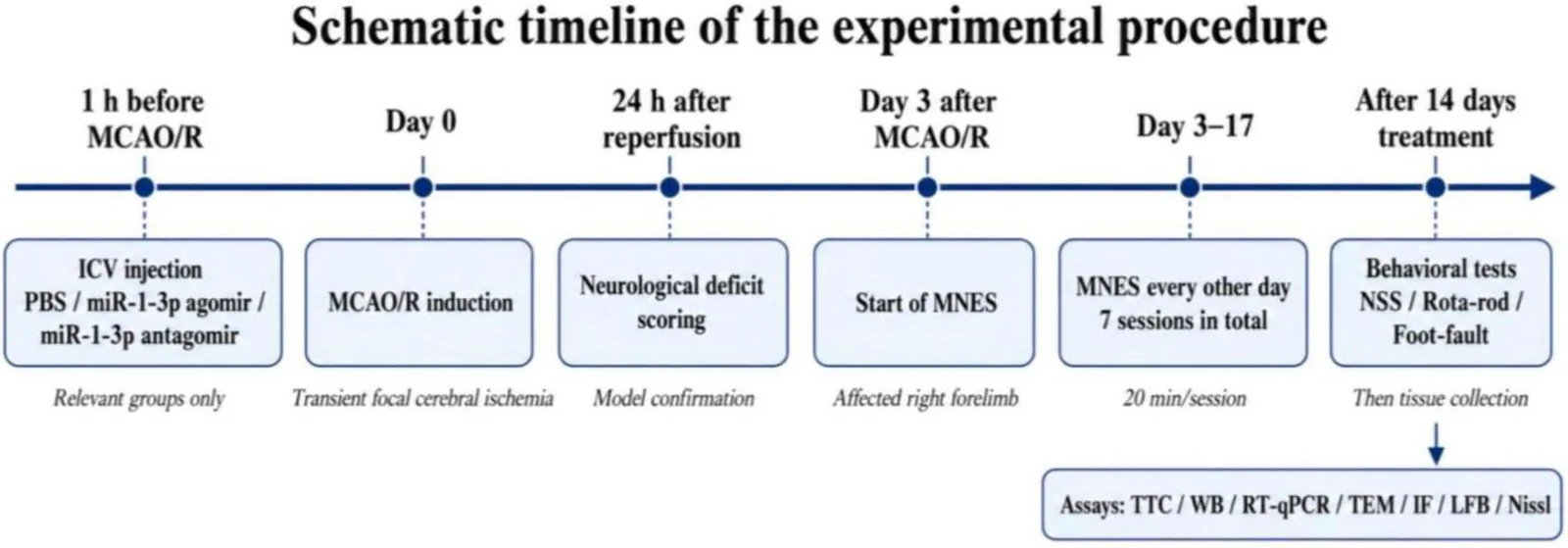
Schematic timeline of the experimental procedure. ICV, intracerebroventricular; PBS, phosphate-buffered saline; miR-1-3p, microRNA-1-3p; MCAO/R, middle cerebral artery occlusion/reperfusion; MNES, median nerve electrical stimulation; NSS, neurological severity score; TTC, 2,3,5-triphenyltetrazolium chloride; WB, Western blotting; RT-qPCR, reverse transcription-quantitative polymerase chain reaction; TEM, transmission electron microscopy; IF, immunofluorescence; LFB, Luxol Fast Blue.

Rats in the PBS, miR-1-3p-antago, miR-1-3p-antago + MNES, miR-1-3p-ago, and miR-1-3p-ago + MNES groups received intracerebroventricular injection 1 h before MCAO/R induction. Model success was evaluated 24 h after reperfusion. MNES was initiated on day 3 after successful MCAO/R induction and administered every other day for 14 days, resulting in seven stimulation sessions. After treatment, behavioral assessments were performed, followed by tissue collection for TTC staining, Western blotting, RT-qPCR, TEM, immunofluorescence staining, LFB staining, and Nissl staining (Figure 1).

### 2.3 Establishment of MCAO model

Transient focal cerebral ischemia was induced using the Zea–Longa intraluminal filament method. Rats were anesthetized with isoflurane at an induction concentration of 3–4% and a maintenance concentration of 1.5%–2%, and then secured in a stereotaxic apparatus (Zhongshi Instruments, Beijing, China). After scalp incision and removal of the periosteum, a cranial window measuring approximately 12 × 10 mm^2^ was created over the left cortical territory supplied by the middle cerebral artery for laser speckle contrast imaging. The cranial window was positioned according to surface anatomical landmarks and visualization of the pial vasculature, rather than by a single stereotaxic anteroposterior (AP)/mediolateral (ML)/dorsoventral (DV) coordinate, because it served as an imaging field for cerebral blood flow monitoring rather than as an injection target. The skull was carefully thinned using a dental drill (Ruierwode Life Science and Technology Co., Ltd., Shenzhen, China) until the pial vasculature was clearly visible (Yuan et al., 2015).

Cerebral blood flow (CBF) was monitored using laser speckle contrast imaging (RFLSI III; Ruierwod Life Science and Technology Co., LTD, Shenzhen, China) at baseline and continuously during arterial occlusion and reperfusion. Successful occlusion was defined as a ≥ 60% reduction in CBF from baseline, with the nadir typically reached within 2–3 min after filament insertion. Successful reperfusion was defined as recovery to ≥70% of baseline CBF following filament withdrawal. Animals that failed to meet these criteria were excluded from further analysis.

For vascular access, the ventral neck region was shaved and sterilized, and a midline cervical incision was made to expose the common carotid artery (CCA), external carotid artery (ECA), and internal carotid artery (ICA) (Fernanda Silva et al., 2024). A nylon monofilament (M8508, Changsha Mayue Biotechnology Co., Ltd.; core diameter 0.20 mm, silicone-coated tip length 6 mm, tip diameter 0.35 ± 0.02 mm) was introduced through the ECA advanced into the ICA, and inserted 1.9–2.0 cm from the carotid bifurcation to occlude the origin of the left middle cerebral artery (MCA). Occlusion was maintained for 60 min, after which the filament was completely withdrawn to allow reperfusion (Onufriev et al., 2021).

The Sham group underwent sham surgery without MCAO, while the remaining seven groups all underwent the Zea-Longa MCAO procedure. Neurological function was assessed 24 h after reperfusion using the Zea-Longa scoring system. Model success was confirmed by the presence of Horner’s syndrome, incomplete forelimb extension, and contralateral circling. Only rats with neurological deficit scores of 1–3 were considered to have successful MCAO and were subsequently enrolled for further analysis. Behavioral assessments were recorded using a CCD camera (270XS 11066, Pixel Fly, PCO, Kelheim, Germany) (Li Y. et al., 2023).

All groups requiring left lateral ventricular injection (PBS, miR-1-3p-antago, miR-1-3p-antago+MNES, miR-1-3p-ago, and miR-1-3p-ago+MNES) received the injection 1 h before MCAO induction. Rats were anesthetized with 1.5% isoflurane, and their heads were secured on a stereotaxic instrument. After removing fur and sterilizing the surgical site, a 3 cm incision was made to expose the skull, and the bregma and lambda points were identified. Intracerebroventricular (ICV) injections were performed using a stereotaxic apparatus. Coordinates were determined relative to bregma as follows: anteroposterior (AP), −0.8 mm; mediolateral (ML), +1.5 mm from the midline (right lateral ventricle); dorsoventral (DV), −4.3 mm from the skull surface. A small burr hole was drilled at the target site, and a micropipette was used to deliver 5 μL of PBS, miR-1-3p agonist, or miR-1-3p antagonist at a rate of 1 μL/min. Injection depth and ventricular localization were verified in preliminary experiments using bromophenol blue staining (Uribe Cardenas et al., 2023). After injection, the needle was kept in place for 5 min before removal, and the incision was sutured. Rats were monitored during recovery in a warm environment.

### 2.4 Median nerve electrical stimulation (MNES)

On the third day following successful MCAO induction, rats in the MNES treatment groups (MNES, miR-1-3p-antago + MNES, and miR-1-3p-ago + MNES) received MNES intervention applied to the affected right forelimb. Rats were anesthetized with 1.5% isoflurane (Reword, China). A disposable stainless-steel needle electrode for peripheral nerve stimulation (0.25 mm × 50 mm; Suyun Medical Co., LTD, Jiangsu, China) was inserted between two tendons approximately 5 mm proximal to the wrist joint, at a 45° angle to a depth of ∼1 mm. Correct electrode placement was functionally verified by visible contraction of the thumb and thenar muscles, indicating activation of the target peripheral nerve.

The electrode was connected to a peripheral nerve stimulator (SY-708A, Suyun Medical Co., LTD, Jiangsu, China). MNES was delivered as pulsed electrical stimulation with a constant current intensity of 1 mA, frequency of 2 Hz, and pulse width of 0.2 ms for a continuous duration of 20 min per session. Following stimulation, the electrode insertion site was disinfected, and the animals were returned to their home cages. MNES was administered every other day for 14 consecutive days, resulting in a total of seven stimulation sessions. Rats in the Sham, MCAO, PBS, miR-1-3p-antago, and miR-1-3p-ago groups underwent identical anesthesia and electrode insertion procedures at the same location and depth for 20 min but did not receive electrical stimulation, serving as procedural controls.

To assess the safety of MNES treatment, the median nerve segment around the stimulation site was collected after the final intervention and examined by hematoxylin and eosin (HE) staining. Briefly, nerve tissues were fixed, processed for routine paraffin embedding, sectioned, and stained with hematoxylin and eosin according to a standard histological protocol. The sections were then dehydrated, cleared in xylene, mounted, and examined under a light microscope. General nerve morphology was evaluated, including nerve fiber arrangement, inflammatory cell infiltration, edema, and obvious structural disruption or scar-like changes (Wick, 2019).

### 2.5 Behavioral testing

Following 14 days of treatment, all behavioral and neurological assessments were performed by an investigator blinded to the experimental groups.

Neurological deficits were evaluated using the Neurological Severity Score (NSS) according to a previously described method (Chen et al., 2001). The NSS is an ordinal scale ranging from 0 to 7. Specifically, score 0 indicates no neurological deficit; score 1 indicates contralateral forelimb flexion when the tail is lifted; score 2 indicates decreased resistance to lateral push when placed on a soft surface; score 3 indicates circling toward the contralateral side when pulled by the tail; score 4 indicates spontaneous circling toward the contralateral side during free movement; score 5 indicates inability to walk independently; score 6 indicates absence of spontaneous activity; and score 7 indicates death. Higher NSS scores indicate more severe neurological impairment.

Motor coordination and endurance were assessed using the Rota-rod test following adaptive training prior to MCAO induction, and animals unable to complete the training were excluded. During the selecting process, rats were trained for 3 consecutive days prior to MCAO, with three trials per day at 10, 20, and 30 rpm. Only animals that could stay on the rod for ≥120 s at 30 rpm were included (Cun et al., 2024). During testing, rats were placed on a rotating rod at a constant speed of 30 revolutions per minute for a maximum duration of 5 min, and the latency to fall was recorded. Each animal completed three trials, and the mean value was used for statistical analysis.

Coordinated motor function of the affected right forelimb was further evaluated using the Foot-fault test, as previously described (Cun et al., 2024). Before MCAO induction, rats underwent an adaptive training and screening protocol for 3 consecutive days. Each day, animals completed three trials on the Rotarod at constant speeds of 10, 20, and 30 rpm. Rats that were unable to complete the adaptive training were excluded from further experiments. Only animals that could remain on the rod for at least 120 s at 30 rpm were included for MCAO induction. During post-treatment testing, rats were placed on a rotating rod at a constant speed of 30 rpm for a maximum duration of 5 min, and the latency to fall was recorded. Each animal completed three trials. Forelimb placement was scored using a 0–6 scale: score 0, total miss; score 1, deep slip with fall; score 2, slight slip without fall; score 3, replacement before weight bearing; score 4, correction to an adjacent rung; score 5, partial placement with the wrist or digits; and score 6, correct placement. Higher scores indicated better forelimb coordination. The average score from three trials was used for statistical analysis.

Following behavioral study, all of rats have been sacrificed by deep anesthesia with 4% isoflurane for 5 min and decapitation for subsequent analysis.

### 2.6 Western blotting of BDNF, TrkB, and MAPK

Cortical tissue from the ischemic region within the left middle cerebral artery territory was homogenized with pre-cooled radioimmunoprecipitation assay lysis buffer and centrifuged, and the supernatants were collected for protein concentration analysis using a bicinchoninic acid (BCA) assay kit (Biosharp, China) (Tripathi et al., 2021). Samples were equilibrated with loading buffer, denatured, and separated by sodium dodecyl sulfate polyacrylamide gel electrophoresis (SDS-PAGE). For BDNF, a 12% gel was used, while 10% gels were prepared for TrkB and MAPK. The separated proteins were transferred onto 0.22 μm PVDF membranes, blocked with 5% skim milk, and incubated with primary antibodies: anti-BDNF (1:3000), anti-TrkB (1:1000), or anti-MAPK (1:3000) (Abcam, United Kingdom), along with the internal control, anti-β-actin (1:3000) or anti-GADPH (1:3000) (Abcam, United Kingdom). After overnight incubation at 4 °C, membranes were washed and incubated with secondary antibody (HRP-conjugated goat anti-rabbit IgG, Affinity Biosciences, China, 1:5000, 1 h, room temperature). Protein bands were detected using enhanced chemiluminescence reagents (Beijing Solarbio Technology Co., Ltd., China) and visualized with a chemiluminescence imager.

### 2.7 MicroRNA target prediction and rT-qPCR

The target genes for miR-1-3p were predicted using the TargetScan (see text footnote 1) database. Among the top 100 predicted target mRNAs for miR-1-3p, BDNF is one of the highest-ranked, with a 98% probability of conserved targeting (PCT) and three conserved binding sites in the 3’ untranslated region (3’ UTR) of miR-1-3p, suggesting that BDNF is the potential target gene of miR-1-3p.

The collected ischemic cortical tissues were processed for reverse transcription-quantitative polymerase chain reaction (RT-qPCR) using TriQuick Reagent (Solarbio, China). The tissues were homogenized, followed by incubation with chloroform and centrifugation. The supernatants were collected, mixed with isopropanol, and centrifuged to isolate the RNAs. The resulting pellets were washed and resuspended in RNase-free water. The purity of the RNA (A260/A280 > 2.0) was then confirmed using a NanoDrop spectrophotometer. Reverse transcription of miRNA and mRNA was carried out using the miRcute Plus miRNA First-Strand cDNA Kit (TIANGEN, China) and the TransScript^®^ All-in-One First-Strand cDNA Synthesis SuperMix (TransGen Biotech, China), respectively, to generate cDNA, which was then stored at −20 °C. qPCR analysis was conducted using specific primers, with glyceraldehyde-3-phosphate dehydrogenase (GAPDH) as the reference gene. The primers for BDNF (BDNF Forward: GTG TGA CAG TAT TAG CGA GTG GG; BDNF Reverse: ACG ATT GGG TAG TTC GGC ATT) and GAPDH (GAPDH Forward: CTG GAG AAA CCT GCC AAG TAT G; GAPDH Reverse: GGT GGA AGA ATG GGA GTT GCT) were designed and supplied by Sevier Biotechnology Co., Ltd., China. For miR-1-3p and the reference gene U6, the primers (miR-1-3p Forward: TGG GTA AGG CAC GCG GTG AAT GC; U6 Forward: CTC GCT TCG GCA GCA CA; Reverse: TGG TGT CGT GGA GTC G) were designed and provided by Sangon Biotech, China. qPCR was performed using a two-step amplification method with the miRcute Plus miRNA qPCR Kit (SYBR^®^ Green) (TIANGEN, China) and PerfectStart^®^ Green qPCR SuperMix (TransGen Biotech, China), according to the manufacturers’ protocols.

Relative quantification of mRNA expression was performed using the comparative cycle threshold (Ct) method. For each sample, the Ct value of the target gene was normalized to that of the internal reference gene GAPDH to obtain the ΔCt value (ΔCt = Ct_target−Ct_GAPDH). Relative mRNA expression levels were then calculated using the 2^–Δ^ ^Δ^ ^Ct^ method, with the Sham group serving as the calibrator (ΔΔCt = ΔCt_sample−ΔCt_Sham). Results are expressed as fold changes relative to the Sham group. All RT-qPCR reactions were performed in technical triplicates, and mean ΔCt values were used for statistical analysis.

### 2.8 Tissue viability assessment

Tissue viability was evaluated using triphenyl tetrazolium chloride (TTC) and Nissl staining. For the TTC staining, the cortical tissue sections were incubated with 2% TTC for 15 min at 37 °C, where infarcted areas appeared white, while non-infarcted areas appeared red. The infarct volume was then calculated using Image-Pro Plus software, expressed as a percentage of the volume of the contralateral hemisphere. For Nissl staining, the cortical tissue sections were fixed with 4% paraformaldehyde, rehydrated with ethanol, and stained with Nissl solution for 9 min. After dehydration with ethanol and clearing with xylene, the infarct volume was determined using Image-Pro Plus software, based on the presence of Nissl bodies in the ischemic penumbra.

### 2.9 Multimodal myelin analysis

Multimodal myelin analysis was conducted to assess myelin structure and function using Western blotting of myelin basic protein (MBP), immunofluorescence staining, Luxol Fast Blue (LFB) staining, and transmission electron microscopy (TEM). For Western blot analysis of MBP, proteins were separated on a 12% SDS-PAGE gel and transferred to a membrane. The membrane was then probed with primary anti-MBP antibody (Abcam, United Kingdom; 1:1000) and an anti-β-actin antibody (1:3000), followed by incubation with HRP-conjugated goat anti-rabbit IgG secondary antibody (Affinity Biosciences, China, 1:5000) and visualized using chemiluminescence. Immunofluorescence staining involved fixing brain tissue sections, blocking with 3% bovine serum albumin (BSA), and incubating with primary anti-MBP (Abcam, United Kingdom, 1:200, overnight, 4 °C), IBA1 (Abcam, United Kingdom, 1:1000, overnight, 4 °C), or NeuN (Abcam, United Kingdom,1:2000, overnight, 4 °C); and secondary antibody (Cy3-conjugated goat anti-rabbit IgG, Servicebio Biotechnology Limited, China, 1:200, 1 h, 37 °C). For nuclear visualization, sections were counterstained with 4’,6-diamidino-2-phenylindole (DAPI, Biosharp, China, 5 min, room temperature). Fluorescence intensity was measured in the cerebral cortex, corpus callosum, and hippocampal CA1. LFB staining involved incubating brain tissue sections in 0.1% LFB for 4 h at 65 °C, followed by lithium carbonate differentiation solution (Servicebio, China) for 5 s. Myelin structure was examined using TEM. Cortical tissue from the infarcted region within the left middle cerebral artery territory was dissected into approximately 1 mmł blocks, post-fixed in 1% osmium tetroxide for 2 h at room temperature, dehydrated in ethanol, embedded in resin, and subsequently cut into ultrathin sections. Sections were stained with 2% uranyl acetate for 8 min, followed by 2.6% lead citrate for an additional 8 min. Ultrastructural changes in the sections were observed with TEM at 8000 × magnification.

### 2.10 Statistical analysis

Experimental data were analyzed using GraphPad Prism 10. Data are presented as the mean ± standard error of the mean (Mean ± SEM). Before group comparisons, the Shapiro-Wilk test was used to assess normality, and Levene’s test was used to assess homogeneity of variance. One-way ANOVA was applied only when the data satisfied the assumptions of normality and variance homogeneity. Tukey’s HSD *post-hoc* test was then used for multiple comparisons within each region, and adjusted *p*-values were reported. If these assumptions were violated, the Kruskal-Wallis test followed by Dunn’s multiple-comparison test was used instead. Effect sizes were reported as η^2^ for the overall ANOVA and as Cohen’s d for selected pairwise comparisons. A two-sided *p*-value < 0.05 was considered statistically significant. Statistical significance was indicated as follows: *p < 0.05, **p < 0.01, ***p < 0.001, and ns indicating *p* > 0.05. All measurements were performed by observers blinded to treatment allocation.

## 3 Results

### 3.1 MNES improves neurological function and reduces brain damage in MCAO rats

**FIGURE 2:**
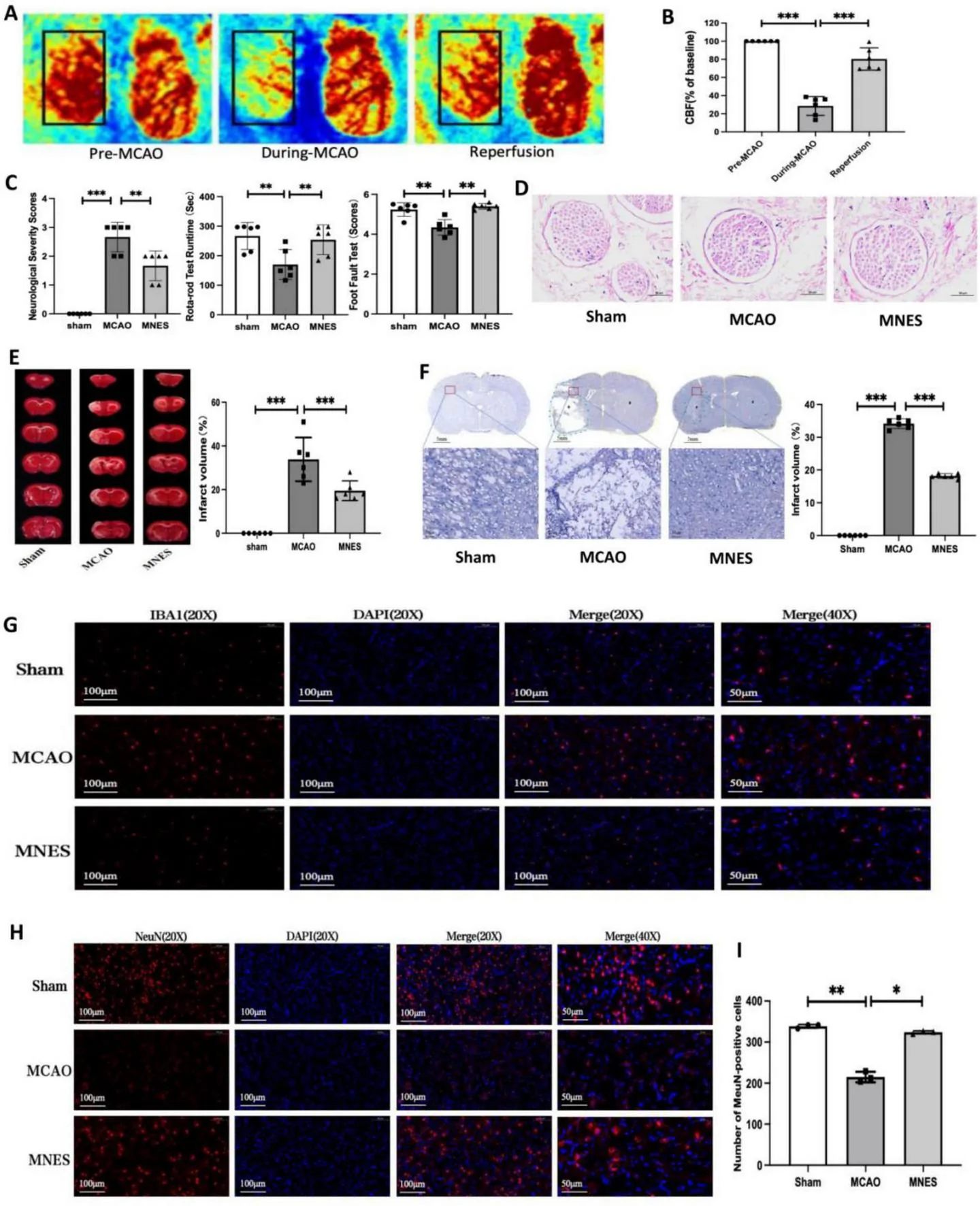
Median nerve electrical stimulation (MNES) improves neurological function and reduces brain damage in middle cerebral artery occlusion (MCAO) rats. **(A)** Representative images of real-time cerebral blood flow monitoring by laser speckle contrast imaging during middle cerebral artery occlusion/reperfusion (MCAO/R). **(B)** Quantification of cerebral blood flow reduction and reperfusion efficacy. **(C)** Neurological behavior scores of neurological severity score (NSS), Rota-rod test, and Foot-fault test. **(D)** Representative images of HE-stained median nerves. **(E)** Representative images of 2,3,5-triphenyltetrazolium chloride (TTC) staining in brain tissue sections and quantification of infarct volume. **(F)** Representative images of Nissl staining in brain tissue sections and quantification of infarct volume. **(G)** Representative Iba-1 Immunofluorescence images of the ischemic penumbra. **(H)** Representative NeuN Immunofluorescence images of the ischemic penumbra. **(I)** Quantitative analysis of neuronal survival. *n* = 6 for each group, except *n* = 3 for each group for immunofluorescence quantifications. *p < 0.05, **p < 0.01, ***p < 0.001.

To determine whether MNES improves functional recovery after ischemic injury, we first evaluated cerebral blood flow, neurological behavior, infarct volume, neuronal injury, and inflammatory changes in MCAO/R rats. Laser speckle contrast imaging confirmed that MCAO/R caused a pronounced reduction in cerebral blood flow within the middle cerebral artery territory during the occlusion phase, followed by restoration of blood flow after filament removal (Figures 2A, B). These hemodynamic changes were accompanied by significant neurological deficits in the MCAO/R rats (Figure 2C). Compared to the MCAO group, MNES treatment significantly improved the recovery of neural function, including reduced NSS scores and increased Rota-rod running time and Foot-fault test scores (*p* < 0.01; Figure 2C). These findings indicate that MNES enhances neurological and motor function following ischemic stroke. However, the behavioral performance of the MNES group did not fully return to the Sham level, indicating that MNES partially, but not completely, restored neurological and motor function after MCAO. To assess the safety of MNES treatment on peripheral nerves, HE staining was performed on the median nerve. The membrane structures in the Sham, MCAO, and MNES groups showed no significant infiltration of inflammatory cells, suggesting that direct MNES application does not cause structural damage to the median nerve (Figure 2D). Ischemic infarct volume was measured using TTC staining. Rats in the MCAO group exhibited significant infarct areas, whereas MNES treatment reduced these pale regions, indicating decreased brain damage and increased tissue viability (*p* < 0.001; Figure 2E). Nissl staining further demonstrated that MCAO caused severe neuronal injury, characterized by reduced Nissl bodies and increased nuclear condensation. MNES treatment alleviated this damage, improving neuronal density and structure (*p* < 0.001; Figure 2F). Immunofluorescence analysis using Iba-1 demonstrated that MCAO induced pronounced microglial activation, as evidenced by increased cell density and morphological transformation toward an activated phenotype. In contrast, MNES treatment significantly attenuated this overactivation (Figure 2G), indicating a modulatory effect on post-stroke neuroinflammation. Furthermore, NeuN staining revealed a marked reduction in neuronal survival within the penumbra following MCAO, whereas MNES intervention significantly increased the number of viable NeuN-positive neurons compared with the MCAO group (*p* < 0.05; Figures 2H,I). Overall, these findings indicate that MNES effectively reduced stroke-induced brain damage and promoted the recovery of neural function.

### 3.2 MNES promotes myelin regeneration in MCAO rats

**FIGURE 3:**
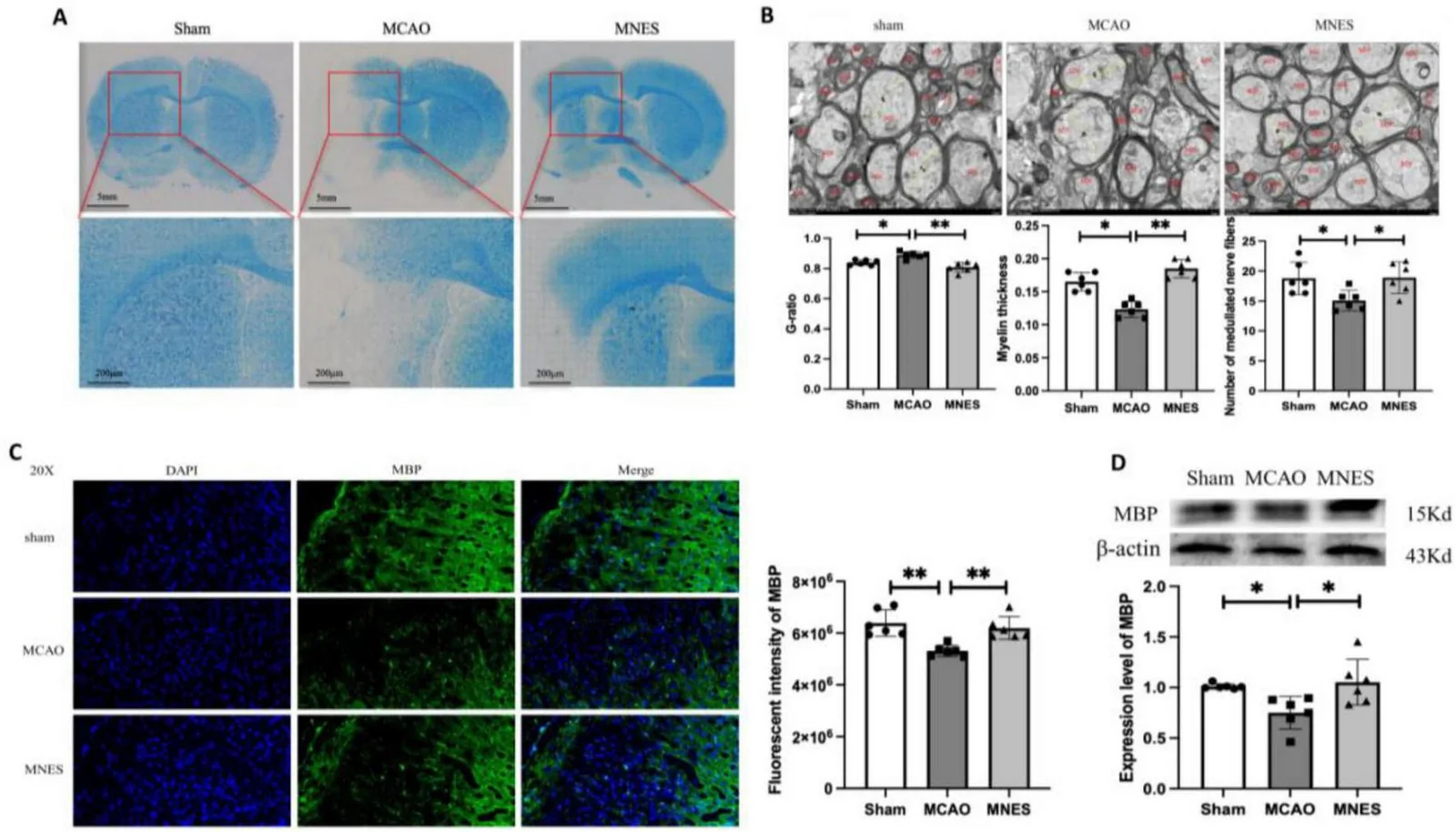
Median nerve electrical stimulation (MNES) promotes myelin regeneration in middle cerebral artery occlusion (MCAO) rats. **(A)** Representative images of brain tissue sections stained with Luxol Fast Blue (LFB). **(B)** Ultrastructure of myelin sheaths and the quantitative analysis of G ratio, myelin sheath thickness, and the number of medullated nerve fibers. **(C)** Representative images of immunofluorescence staining and quantitative analysis of myelin basic protein (MBP). **(D)** Representative Western blot bands and quantitative analysis of MBP. *n* = 6 for each group. *p < 0.05, **p < 0.01.

Because myelin integrity is essential for neural conduction and post-stroke functional recovery, we next examined whether MNES affected myelin preservation and repair using LFB staining, TEM, MBP immunofluorescence, and Western blotting. Myelin regeneration plays a crucial role in post-stroke rehabilitation. To assess the extent of myelin repair, myelin integrity was evaluated using LFB staining, TEM, and immunofluorescence and Western blot of MBP expression. LFB staining showed better-preserved myelin in the MNES group, indicating reduced myelin loss rather than active preservation (Figure 3A). In addition, TEM analysis showed increased myelin sheath thickness with a lower G-ratio defined as axon diameter/(axon + myelin sheath diameter) and a higher density of myelinated fibers in the MNES group compared to the MCAO group (*p* < 0.01; Figure 3B). Immunofluorescence staining of brain tissues demonstrated elevated MBP expression, with stronger green fluorescence observed in the cerebral cortex of MNES-treated animals (*p* < 0.01; Figure 3C). Consistently, Western blot analysis confirmed a significant increase in MBP protein levels in the MNES group compared to the MCAO group (*p* < 0.05; Figure 3D). These findings suggest that MNES effectively promotes myelin repair and recovery following ischemic stroke.

### 3.3 MNES up-regulates BDNF, TrkB, and MAPK expression in MCAO rats

**FIGURE 4:**
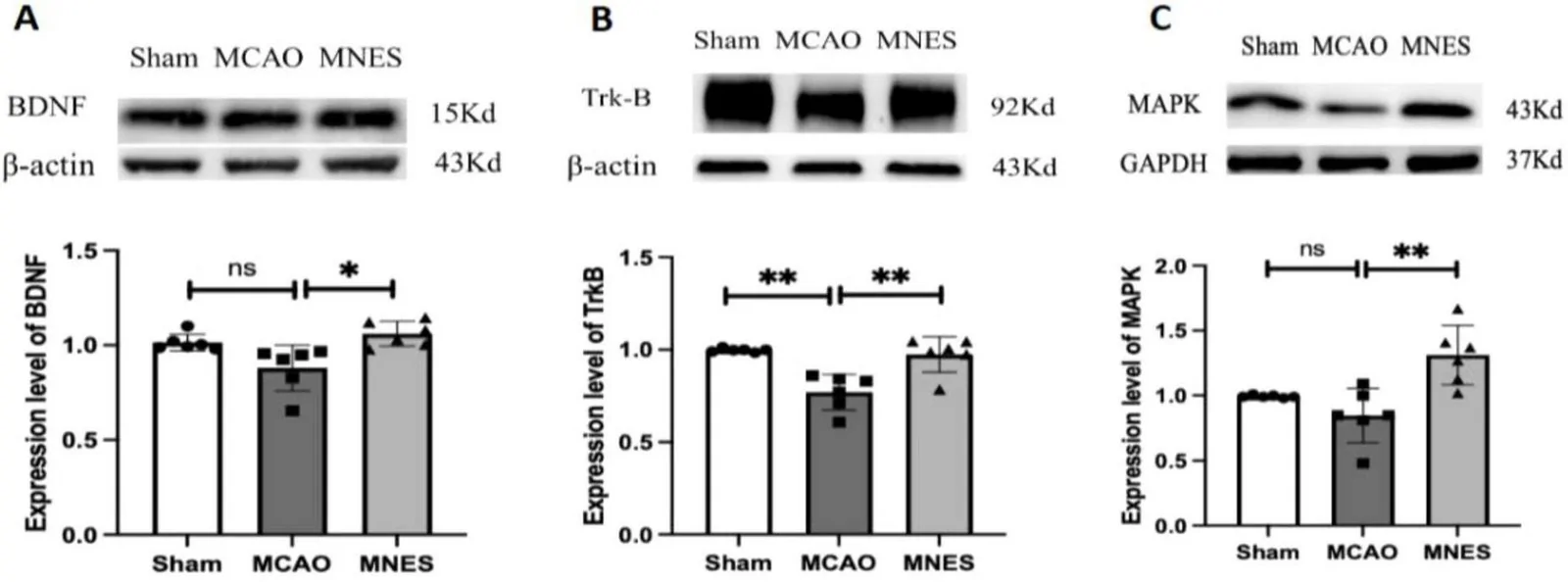
Median nerve electrical stimulation (MNES) up-regulates brain-derived neurotrophic factor (BDNF), tropomyosin receptor kinase B (TrkB), and mitogen-activated protein kinase (MAPK) expression in middle cerebral artery occlusion (MCAO) rats. Representative Western blot bands and quantitative analysis of panels **(A)** BDNF, **(B)** TrkB, and **(C)** MAPK. *n* = 6 for each group. *p < 0.05, **p < 0.01. Original Western blot images are available in the Supplementary material.

To explore molecular changes that may be associated with MNES-related myelin recovery, we measured the expression of BDNF, TrkB, and MAPK in the ischemic cortical tissue. Western blot analysis showed that BDNF protein levels were slightly reduced in the MCAO group, although this difference was not significant (*p* > 0.05), and were significantly increased following MNES treatment (*p* < 0.05; Figure 4A). TrkB expression was significantly decreased in the MCAO group (*p* < 0.01) and was modestly but significantly increased after MNES treatment (*p* < 0.01; Figure 4B). Although MAPK levels showed a slight, non-significant decrease in the MCAO group (*p* > 0.05), they were significantly elevated in the MNES group (*p* < 0.01; Figure 4C). These findings indicate that MNES treatment was associated with increased expression of BDNF, TrkB, and MAPK in MCAO rats.

### 3.4 MNES up-regulates BDNF, TrkB, and MAPK expression through inhibiting miR-1-3p level

**FIGURE 5:**
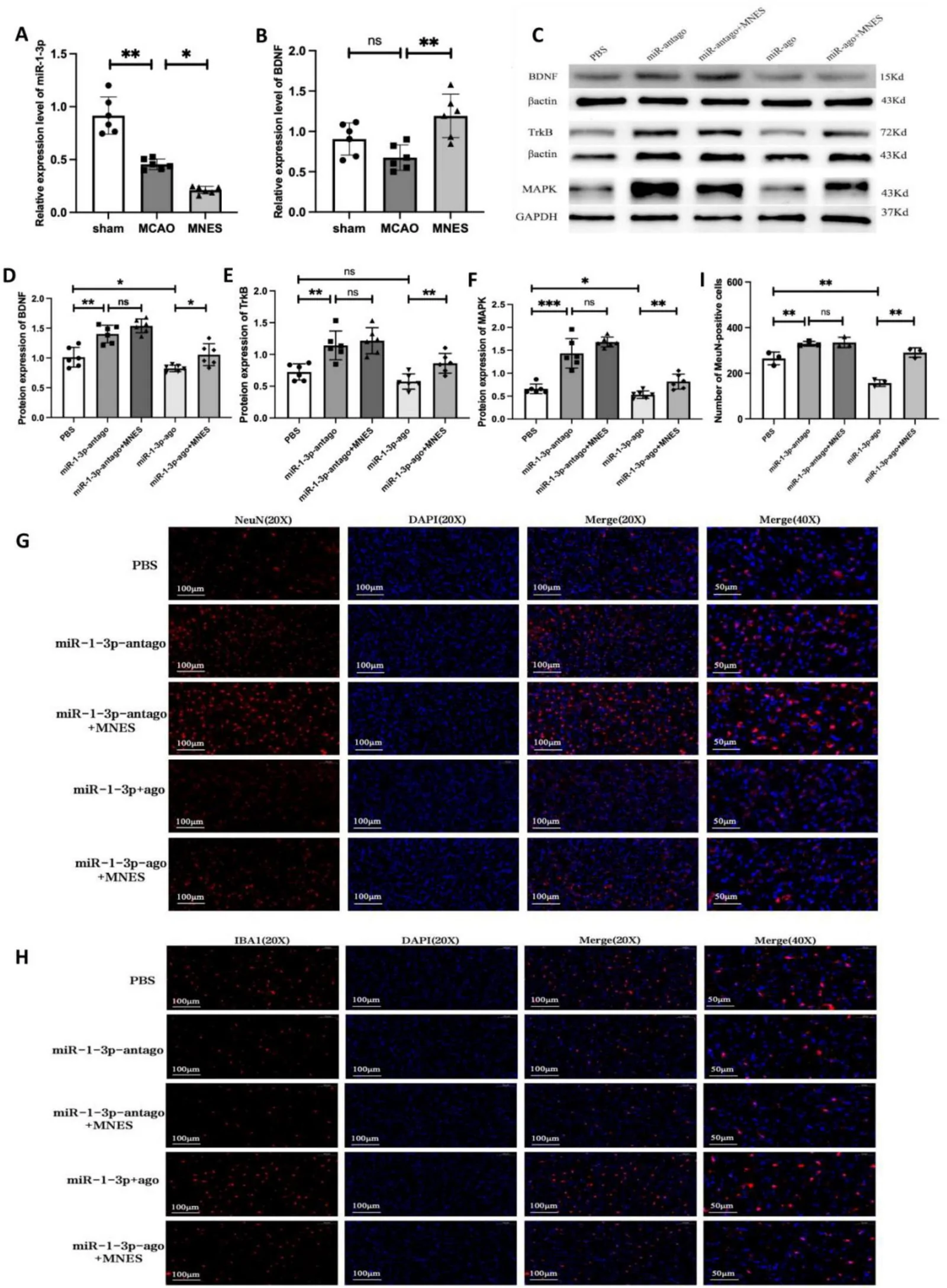
Median nerve electrical stimulation (MNES) up-regulates brain-derived neurotrophic factor (BDNF), tropomyosin receptor kinase B (TrkB), and mitogen-activated protein kinase (MAPK) expression through inhibiting miR-1-3p level. Reverse transcription-quantitative polymerase chain reaction (RT-qPCR) analysis for panels **(A)** miR-1-3p and **(B)** BDNF mRNA expression levels. Representative Western blot bands **(C)** and quantitative analysis of panels **(D)** BDNF, **(E)** TrkB, and **(F)** MAPK. **(G)** Representative Iba-1 Immunofluorescence images of the ischemic penumbra. **(H)** Representative NeuN Immunofluorescence images of the ischemic penumbra. **(I)** Quantitative analysis of neuronal survival. For RT-qPCR and Western blot, *n* = 6 for each group; for immunofluorescence quantifications, *n* = 3 for each group. *p < 0.05, **p < 0.01, ***p < 0.001. Original Western blot images are available in the Supplementary material.

Since bioinformatic prediction suggested BDNF as a potential target of miR-1-3p, we further examined whether changes in miR-1-3p expression were associated with BDNF/TrkB-MAPK-related molecular responses after MCAO. All rats in these experiments (except the Sham group) were subjected to MCAO/R as described in Methods. PBS group served as the vehicle control for intracerebroventricular injection, whereas the miR-1-3p-antago and miR-1-3p-ago groups were used to evaluate the effects of miR-1-3p inhibition or activation under ischemic conditions. RT-qPCR analysis showed that miR-1-3p expression in the cerebral cortex was lower in the MCAO group compared to the Sham group (*p* < 0.01) and was further reduced following MNES treatment (*p* < 0.05; Figure 5A). BDNF, identified as a potential target of miR-1-3p, exhibited a non-significant decrease in the MCAO group (*p* > 0.05), but was significantly upregulated in the MNES group (*p* < 0.01; Figure 5B). Although BDNF is a predicted target of miR-1-3p, the inverse correlation was not strict across all groups, possibly due to post-transcriptional regulation, additional miRNA interactions, or tissue-specific differences in miRNA-mRNA pairing efficiency. These findings suggest that MNES treatment leads to further downregulation of miR-1-3p expression after stroke, which may be linked to the upregulation of BDNF in the cerebral cortex.

In experiments involving miR-1-3p modulation, BDNF levels increased significantly in the miR-1-3p antagonist group (*p* < 0.001) and decreased in the agonist group (*p* < 0.05) compared to PBS control group, suggesting that BDNF expression is regulated by miR-1-3p. Furthermore, Western blot analysis showed that the miR-1-3p-antago significantly upregulated the expression of BDNF (*p* < 0.01), TrkB (*p* < 0.01), and MAPK (*p* < 0.001), while the miR-1-3p-ago significantly reduced BDNF (*p* < 0.05) and MAPK (*p* < 0.05) expression (Figures 5C–F). Consistently, Immunofluorescence analysis revealed that miR-1-3p-antago suppressed microglial overactivation (Iba-1) and enhanced neuronal survival (NeuN, *p* < 0.01) in the ischemic penumbra, whereas miR-1-3p-ago produced the opposite effects, thereby reversing the protective benefits of MNES (*p* < 0.01) (Figures 5G–I). Overall, these findings support the regulatory role of miR-1-3p in the BDNF/TrkB-MAPK signaling pathway and highlight its potential as a therapeutic target for stroke recovery.

### 3.5 Effects of MNES under miR-1-3p modulations

**FIGURE 6:**
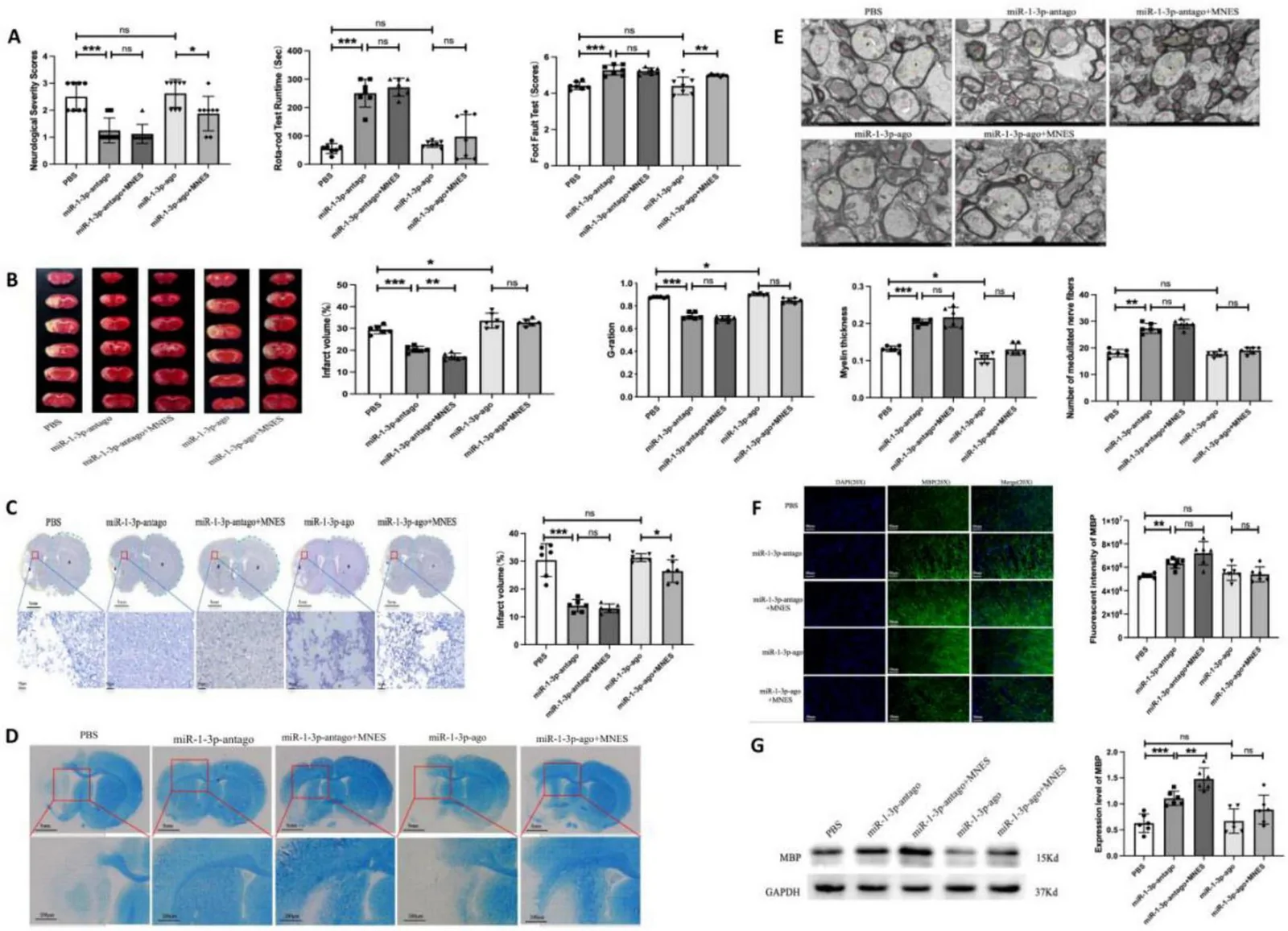
Myelin regeneration and neuroprotection of median nerve electrical stimulation (MNES) were eliminated by the activation of miR-1-3p. **(A)** Neurological behavior scores of Neurological Severity Score (NSS), Rota-rod test, and Foot-fault test. **(B)** Representative images of triphenyl tetrazolium chloride (TTC) staining in brain tissue sections and quantification of infarct volume. **(C)** Representative images of Nissl staining in brain tissue sections and quantification of infarct volume. **(D)** Representative images of brain tissue sections stained with Luxol Fast Blue (LFB). **(E)** Ultrastructure of myelin sheaths and the quantitative analysis of G ratio, myelin thickness, and the number of medullated nerve fibers. **(F)** Representative images of immunofluorescence staining and quantitative analysis of myelin basic protein (MBP). **(G)** Representative Western blot bands and quantitative analysis of MBP. *n* = 6 for each group. *p < 0.05, **p < 0.01, ***p < 0.001. Original Western blot images are available in the Supplementary material.

To further examine whether miR-1-3p participates in MNES-related neuroprotection and myelin repair, we evaluated behavioral outcomes, infarct volume, myelin structure, and MBP expression after miR-1-3p inhibition or activation. All rats in these experiments (except the Sham group) were subjected to MCAO/R as described in Methods. Treatment with the miR-1-3p-antago significantly improved behavioral performance (NSS, RR, and FFT: *p* < 0.001; Figure 6A), reduced infarct volume percentage (TTC staining: *p* < 0.001; Figure 6B and Nissl staining: *p* < 0.001; Figure 6C), and enhanced myelin regeneration, as shown by increased preserved myelin under LFB staining (Figure 6D), improved myelin structure (TEM analysis of G-ratio, myelin thickness and medullated fiber count: *p* < 0.001; Figure 6E), and increased MBP expression (Immunofluorescence in the cerebral cortex: *p* < 0.001; Figure 5F and Western blot: *p* < 0.001; Figure 6G). These findings suggest that inhibiting miR-1-3p activity confers neuroprotection comparable to that observed with MNES treatment. In contrast, activation of miR-1-3p with an agonist produced mostly non-significant effects (*p* > 0.05).

Following MNES treatment, the miR-1-3-ago+MNES group showed significant improvement in behavioral tests (NSS: *p* < 0.05 and FFT: *p* < 0.01; Figure 6A) and reduction in infarct volume (Nissl staining: *p* < 0.05; Figure 6C). However, no significant improvements were observed in myelin structure (*p* > 0.05), suggesting that although MNES may mitigate some adverse effects of the agonist, it does not fully reverse the damage. In contrast, the miR-1-3p-antago+MNES group showed mostly non-significant changes (*p* > 0.05), except for TTC staining (*p* < 0.01; Figure 6B) and MBP expression by Western blotting (*p* < 0.01; Figure 6G). Overall, the combination of miR-1-3p antagonist and MNES demonstrated the most substantial neuroprotective effects, suggesting that downregulation of miR-1-3p, in combination with MNES, may offer greater therapeutic benefit following stroke.

## 4 Discussion

Stroke causes significant myelin damage, disrupting neural communication and leading to functional deficits. This study demonstrated the therapeutic effects of MNES on ischemic stroke in rats, focusing on the role of miR-1-3p and its regulation of the BDNF/TrkB-MAPK pathway in promoting myelin regeneration. The results showed that MNES downregulates miR-1-3p, which in turn activates the BDNF/TrkB-MAPK signaling pathway. Additionally, the use of a miR-1-3p antagonist led to enhanced myelin repair and functional recovery, highlighting the importance of miR-1-3p in neural repair. These findings help explain the neuroprotective effects of MNES and suggest new therapeutic possibilities for stroke recovery. Despite the application of invasive MNES in this study, no scarring of median nerve fibers or increase in immune cells was observed, as indicated by the absence of increased pink-stained collagen fibers and the lack of additional purple-stained nuclei, suggesting the safety of MNES.

Bioinformatics plays a vital role in understanding the regulatory mechanisms of miRNAs by predicting their targets and annotating their functions. In this study, TargetScan analysis predicted the interaction between miR-1-3p and BDNF mRNA, revealing a strong binding affinity of miR-1-3p to the 3’ UTR of BDNF, suggesting it as a potential target. This finding aligns with previous studies, which demonstrated that miR-1-3p directly targets the 3’ UTR of BDNF, downregulating its expression, and validated this interaction using a luciferase reporter assay (Xie et al., 2023). Consistent with these findings, our results showed that MNES significantly reduced miR-1-3p levels, increased BDNF expression, and activated the downstream TrkB-MAPK signaling pathway, promoting myelin regeneration. This observation was further validated using miR-1-3p antagonist and agonist.

Several studies have demonstrated the role of electrical stimulation in regulating miRNA activity during neural processes. For example, electroacupuncture increases miR-223-3p expression to inhibit neuronal autophagy, indicating that neural electrical stimulation can modulate miRNA levels and impact cellular functions like autophagy and apoptosis (Zou et al., 2021). Similarly, electrical stimulation has been shown to influence miR-363-5p, promoting neurite outgrowth through its target, DCLK1 (Quan et al., 2017). In epilepsy models, deep brain stimulation was found to alter miRNA levels, including miR-1-3p, which is associated with myelin regeneration and neuroprotection (Costard et al., 2019). Our study aligns with these findings, showing that MNES reduces miR-1-3p levels while increasing BDNF to support myelin regeneration in ischemic stroke. Electrical stimulation can enhance BDNF expression through mechanisms like calcium/calmodulin-dependent protein kinase (CaMK) pathway activation, regulation of the serotonergic system, and synaptic activity enhancement (Yan et al., 2016). These processes contribute to myelin repair and neuronal survival.

In this study, Western blot results indicated increased BDNF, TrkB, and MAPK expression following MNES treatment, suggesting that miR-1-3p downregulation activates the BDNF/TrkB-MAPK pathway. BDNF’s interaction with the TrkB receptor activates pathways like PI3K/Akt, MAPK/ERK, and PLCγ, which are critical for neuronal growth and survival. The MAPK/ERK pathway, in particular, plays a vital role in ischemic stroke recovery by promoting neuronal survival and differentiation (Wang et al., 2024; Yoshii and Constantine-Paton, 2010). The activation of the TrkB-MAPK pathway, triggered by increased BDNF levels, plays a critical role in promoting myelin regeneration after ischemic stroke. This pathway enhances cellular processes such as proliferation, differentiation, and survival, which are essential for myelin repair (Fletcher et al., 2018; Wang et al., 2024). The effects observed in our study, including reduced brain infarct volume and improved myelin structure in MNES-treated rats, support the idea that activating this pathway promotes myelin regeneration, axonal repair, and synaptic connectivity, which enhance neuronal communication and lead to improved motor control and overall coordination (Schirò et al., 2022).

Myelin regeneration involves oligodendrocyte precursor cells (OPCs) maturing into oligodendrocytes, a process regulated by BDNF binding to TrkB receptors on these cells, leading to activation of the MAPK pathway and promoting myelin formation around active axons (Bradl and Lassmann, 2010; Katan and Luft, 2018). This interaction ensures precise coordination between neuronal activity and myelination (Huntemer-Silveira et al., 2020). Additionally, the TrkB-MAPK pathway helps protect oligodendrocytes from apoptosis, maintaining the cell population needed for continuous repair (Fletcher et al., 2018). Oligodendrocytes produce myelin basic protein (MBP) in key regions such as the cerebral cortex, corpus callosum, and hippocampus CA1 region, which is crucial for restoring myelin integrity and distribution, as evidenced by LFB staining. Following this, structural refinement occurs as the newly formed myelin undergoes compaction and thickening (Choe et al., 2023). This process significantly enhances myelin sheath thickness, uniformity, and the number of myelinated neurons, which are crucial for the proper functioning of myelinated axons. The increased compaction of myelin layers ensures efficient signal transmission along the axons, thereby reducing the likelihood of neurological deficits and promoting regeneration and functional recovery (Mitew et al., 2014). Given its role in myelin regeneration, the TrkB-MAPK pathway presents a promising therapeutic target for enhancing recovery post-stroke. Strategies such as modulating BDNF levels or directly activating TrkB receptors could accelerate remyelination and improve outcomes.

In the present study, administration of the miR-1-3p antagonist to MCAO rats resulted in significant improvements across multiple outcome measures, supporting a functional role of miR-1-3p inhibition in post-stroke recovery. These effects were accompanied by activation of the BDNF/TrkB–MAPK signaling pathway, improved neurological performance, and enhanced indices of myelin repair, suggesting that suppression of miR-1-3p contributes to neuroprotection and white matter preservation. Notably, when miR-1-3p antagonism was combined with MNES, additional improvements were observed in some parameters; however, most did not reach statistical significance. One possible explanation is that miR-1-3p antagonism alone may already approach a near-maximal therapeutic effect, thereby limiting the capacity for MNES to produce further measurable gains.

This observation is consistent with the concept of a ceiling effect in stroke recovery, whereby therapeutic efficacy plateaus despite increased intervention intensity or combination strategies. Such ceiling effects have been reported in various neurorehabilitative interventions and represent a major challenge in optimizing post-stroke treatment outcomes (Parente, 2016). While some evidence suggests that prolonged or repeated interventions may overcome this limitation (Petriv et al., 2024), extended stimulation must be approached cautiously due to potential risks of peripheral nerve or muscle injury (Uçar et al., 2024). Future work may therefore benefit from exploring combinatorial strategies that integrate MNES with conventional rehabilitation, pharmacological agents, or robotic-assisted therapies, which may synergistically enhance neuroplasticity and functional recovery (Dai et al., 2024; Szelenberger et al., 2020).

In contrast, administration of the miR-1-3p agonist alone did not significantly exacerbate ischemic injury compared with the PBS control in most outcome measures. This finding may reflect the severity of the initial ischemic insult induced by MCAO, which could limit the detectability of additional injury. Moreover, endogenous repair mechanisms activated after stroke, such as angiogenesis, neurogenesis, and synaptogenesis, may partially buffer against further damage, thereby masking the detrimental effects of miR-1-3p overexpression (Liu et al., 2020; Zheng et al., 2022). Importantly, when MNES was applied in the presence of miR-1-3p agonist, MNES significantly reduced miR-1-3p levels and reactivated the BDNF/TrkB–MAPK pathway, resulting in improved neuronal function and increased Nissl body density, indicative of enhanced neuronal health.

Despite these beneficial effects, myelin-related outcomes did not show significant improvement in the miR-1-3p-ago + MNES group. This discrepancy suggests that while MNES can rapidly modulate neuronal survival and functional recovery, myelin regeneration may require a longer time course to manifest. Persistent miR-1-3p overexpression may prolong white matter injury, delaying oligodendrocyte-mediated repair processes (Li et al., 2021). Consistent with this interpretation, myelin regeneration is known to be a slow and multistage process, involving early OPCs recruitment followed by differentiation into mature myelinating oligodendrocytes over weeks to months (Huang et al., 2023; Lachapelle et al., 2005). Thus, the observation period in the present study may have been insufficient to capture the full extent of MNES-induced myelin repair, underscoring the need for longer-term studies.

The translational relevance of this study should be interpreted with caution. First, only adult male rats were used, and therefore the present findings do not address potential sex-related differences in ischemic injury, myelin repair, neurotrophic signaling, or responsiveness to MNES. Second, the MCAO/R model provides a controlled and reproducible experimental platform, but it does not fully reproduce the heterogeneity of human ischemic stroke, including differences in age, vascular risk factors, comorbidities, infarct location, infarct volume, stroke severity, medication use, and rehabilitation background. Third, although MNES improved neurological scores and motor-related behavioral outcomes in rats, these endpoints cannot be directly equated with clinically meaningful recovery in human patients. Therefore, the current findings should be regarded as preclinical evidence supporting the potential biological relevance of MNES, rather than direct evidence of clinical efficacy. Future studies should include both sexes, aged animals, comorbidity models, longer follow-up periods, and clinically aligned functional assessments. Ultimately, well-designed clinical studies are required to determine whether MNES-associated myelin repair and pathway modulation translate into meaningful functional recovery in patients after stroke.

It should be noted that although MNES improved behavioral outcomes compared with untreated MCAO rats, the MNES group still showed residual differences from the Sham group in Figure 2C. This finding indicates that MNES alleviates, rather than completely reverses, ischemia-induced neurological dysfunction within the current treatment window. Therefore, the therapeutic effect of MNES should be interpreted as a partial functional recovery after stroke, and longer intervention periods or combined rehabilitation strategies may be required to achieve more complete behavioral restoration.

## 5 Conclusion

This study provides evidence that MNES may serve as a promising neuromodulatory strategy for promoting early myelin repair-related changes and functional recovery after ischemic stroke. In the rat MCAO model, MNES-treated animals showed improved motor performance, reduced infarct volume, and better preservation of white matter integrity. At the molecular level, MNES treatment was accompanied by downregulation of miR-1-3p, upregulation of BDNF expression, and increased activity of the downstream TrkB–MAPK signaling pathway. The inhibitor-based experiments further supported the possible involvement of the miR-1-3p/BDNF/TrkB–MAPK-related pathway in MNES-mediated neuroprotection and myelin repair. Together, these findings suggest that MNES may have therapeutic potential as an adjunct intervention for ischemic stroke. They also provide a rationale for future studies examining whether similar neuromodulatory strategies could be relevant to other neurological conditions involving white matter injury, such as traumatic brain injury and selected neurodegenerative disorders. However, because the present study was conducted in a rodent model and involved a relatively short observation period, direct clinical translation remains limited. Future studies should evaluate the durability of MNES-mediated benefits, further verify the causal role of the miR-1-3p/BDNF/TrkB–MAPK axis using additional gain- and loss-of-function approaches, investigate other downstream pathways such as PI3K and PLCγ, and assess combinatorial treatment strategies to refine the therapeutic potential of MNES.

## Data Availability

The original contributions presented in this study are included in this article/Supplementary material, further inquiries can be directed to the corresponding authors.

## Footnotes

1
www.targetscan.org
