# Supplementary material for: Median nerve electrical stimulation promotes myelin regeneration through regulating miR-1-3p/BDNF/TrkB-MAPK pathway in a transient cerebral ischemia rat model
Source: Front Behav Neurosci. 2026 Sep 11;20:1820913. doi: 10.3389/fnbeh.2026.1820913 (PMC13612498; doi:10.3389/fnbeh.2026.1820913)
Supplement: Supplementary file 1 [file Data_Sheet_1.docx]

Supplementary Material

# 1 Supplementary Data

| Western Blot | | |
| --- | --- | --- |
| Figure 2D: MBP | | |
| No. | 1-3 | |
|  |  | |
| MBP |  | |
| β-actin |  | |
| MBP |  | |
| β-actin |  | |
| No. | 4-6 | |
|  |  | |
| MBP |  | |
| β-actin |  | |
| MBP |  | |
| β-actin |  | |
| Figure 3A: BDNF | | |
| No. | 1-2 | |
|  |  |  |
| BDNF |  |  |
| β-actin |  |  |
| No. | 3-4 | |
|  |  |  |
| BDNF |  |  |
| β-actin |  |  |
| No. | 5-6 |  |
|  |  |  |
| BDNF |  |  |
| β-actin |  |  |
| Figure 3B: Trk-B | | |
| No. | 1-2 |  |
|  |  |  |
| Trk-B |  |  |
| β-actin |  |  |
| No. | 3-4 |  |
|  |  |  |
| Trk-B |  |  |
| β-actin |  |  |
| No. | 5-6 |  |
|  |  |  |
| Trk-B |  |  |
| β-actin |  |  |
|  | Figure 3C: MAPK | |
| No. | 1-3 | |
|  |  | |
| MAPK |  | |
| GAPDH |  | |
| MAPK |  | |
| GAPDH |  | |
| No. | 4-6 | |
|  |  | |
| MAPK |  | |
| GAPDH |  | |
| MAPK |  | |
| GAPDH |  | |
|  | | |
| Figure 4C | | |
| Figure 4C: BDNF | | |
| No.1-2 |  |  |
| BDNF |  |  |
| β-actin |  |  |
| No.3-4 |  |  |
| BDNF |  |  |
| β-actin |  |  |
| No.5-6 |  |  |
| BDNF |  |  |
| β-actin |  |  |
|  | | |
| Figure 4C: Trk-B | | |
| No.1-2 |  |  |
| Trk-B |  |  |
| β-actin |  |  |
| No.3-4 |  |  |
| Trk-B |  |  |
| β-actin |  |  |
| No.5-6 |  |  |
| Trk-B |  |  |
| β-actin |  |  |
|  | | |
| Figure 4C: MAPK | | |
| No.1-2 |  |  |
| MAPK |  |  |
| GAPDH |  |  |
| No.3-4 |  |  |
| MAPK |  |  |
| GAPDH |  |  |
| No.5-6 |  |  |
| MAPK |  |  |
| GAPDH |  |  |
|  | | |
| Figure 5G: MBP | | |
| No.1-2 |  |  |
| MBP |  |  |
| β-actin |  |  |
| No.3-4 |  |  |
| MBP |  |  |
| β-actin |  |  |
| No.5-6 |  |  |
| MBP |  |  |
| β-actin |  |  |
